# The Korean Clinical Research Center for End-Stage Renal Disease Study Validates the Association of Hemoglobin and Erythropoiesis-Stimulating Agent Dose with Mortality in Hemodialysis Patients

**DOI:** 10.1371/journal.pone.0140241

**Published:** 2015-10-09

**Authors:** Owen Kwon, Hye Min Jang, Hee-Yeon Jung, Yon Su Kim, Shin-Wook Kang, Chul Woo Yang, Nam-Ho Kim, Ji-Young Choi, Jang-Hee Cho, Chan-Duck Kim, Yong-Lim Kim, Sun-Hee Park, Clinical Research Center for End-Stage Renal Disease (CRC- ESRD) Investigators

**Affiliations:** 1 Department of Internal Medicine, Kyungpook National University School of Medicine, Daegu, Korea; 2 Department of Statistics, Kyungpook National University, Daegu, Korea; 3 Department of Internal Medicine, Seoul National University College of Medicine, Seoul, Korea; 4 Department of Internal Medicine, Yonsei University College of Medicine, Seoul, Korea; 5 Department of Internal Medicine, The Catholic University of Korea College of Medicine, Seoul, Korea; 6 Department of Internal Medicine, Chonnam National University Medical School, Gwangju, Korea; 7 Clinical Research Center for End-Stage Renal Disease in Korea, Daegu, Korea; The University of Tokyo, JAPAN

## Abstract

**Background:**

Anemia is an important risk factor for mortality in hemodialysis (HD) patients. However, higher hemoglobin (Hb) is not necessarily better, as seen in several studies. This study aimed to validate the clinical use of an Hb target of 10–11 g/dL in Korean HD patients.

**Methods:**

A total of 1,276 HD patients from the Clinical Research Center (CRC) for End-Stage Renal Disease (ESRD) were investigated in a prospective observational study. Cox proportional hazard analysis was conducted for each category of time-dependent Hb level and erythropoiesis-stimulating agent (ESA) dose, with subgroup analysis stratified by age and diabetes status.

**Results:**

Using a reference Hb level of 10–11 g/dL, the hazard ratios (HRs) of death were 5.12 (95% confidence interval [CI], 2.62–10.02, P <0.05) for Hb level <9.0 g/dL, and 2.03 (CI, 1.16–3.69, P <0.05) for Hb level 9.0–10.0 g/dL, after adjustment for multiple clinical variables. However, an Hb level ≥11 g/dL was not associated with decreased mortality risk. In an adjusted model categorized by Hb and ESA dose, the risk of death at an Hb level <10 g/dL and a higher dose of ESA (≥126 U/kg/week) had an HR of 2.25 (CI, 1.03–4.92, P <0.05), as compared to Hb level 10–11 g/dL and a lower dose of ESA. In subgroup analysis, those older than 65 years or who were diabetic had greater risk for mortality only in Hb category <9.0 g/dL. However, there was no significant interaction between age or diabetes status and Hb.

**Conclusion:**

Using CRC-ESRD data, we validated the association between Hb and ESA dose and mortality in Korean HD patients. The clinical practice target of an Hb of 10–11 g/dL before the new KDIGO guideline era seems reasonable considering its survival benefit in HD patients.

## Introduction

Anemia develops in nearly all patients with advanced chronic kidney disease (CKD) [1]. Erythropoiesis-stimulating agents (ESAs) have been widely used as a major treatment option for renal anemia since the US Food and Drug Administration approved synthetic erythropoietin in 1989. Several observational studies have shown that severe anemia in hemodialysis (HD) patients is related to increased morbidity and mortality [2–5]. However, randomized controlled trials (RCTs) in patients with CKD have demonstrated that using an ESA with a higher target hemoglobin (Hb) level provided no additional benefits; rather, it was associated with an increased risk of adverse vascular events including hypertension, stroke, and vascular thrombosis [6–10]. Based on these RCTs, recent guidelines advise starting ESA treatment at an Hb level < 10 g/dL and reducing or interrupting the dose at an Hb level ≥ 11 g/dL [11]. However, this does not necessarily mean that an Hb level of 10–11 g/dL is the most appropriate target for managing anemia in dialysis patients.

In Korea, owing to limitations in reimbursement policy when using ESA in dialysis patients, the clinical practice target for Hb was adjusted at 10–11 g/dL before the release of the 2012 Kidney Disease Improving Global Outcomes (KDIGO) guidelines. However, whether an Hb level of 10–11 g/dL is a reasonable target or a higher Hb target would be desirable in HD patients remains a concern. Therefore, we aimed to evaluate the association between Hb and mortality in a prospective, observational study in Korea.

Whether the increased risk of mortality in a group with higher Hb target observed in recent RCTs can be attributed to the higher Hb level, higher ESA dose, or both remains unclear. Regarding ESA dose, a recent study suggested that a higher dose of ESA was associated with higher mortality in HD patients [12]. In addition, a decreased Hb level over time is associated with an increased risk of death regardless of baseline Hb; thus, requiring a higher dose of ESA is a surrogate for a higher risk of death [13]. Therefore, we additionally aimed to evaluate whether ESA dose affected mortality risk for different Hb levels in HD patients. Lastly, it is not clear whether patient characteristics including age or diabetes status affect the risk of anemia for mortality. Thus, we aimed to evaluate whether age or diabetes status affect the association of Hb and mortality in subgroup analysis of HD patients from a Korean cohort of the Clinical Research Center (CRC) for End-Stage Renal Disease (ESRD).

## Materials and Methods

### Patients

We collected data from patients undergoing maintenance HD who were participating in the prospective study of the Korean CRC-ESRD. The CRC-ESRD cohort consisted of dialysis patients enrolled from September 1, 2008, at 31 centers in Korea. To be eligible for inclusion in the primary cohort, patients must have been at least 20 years old at the time of enrollment. Patients were excluded if they were planned to undergo kidney transplantation in 3 months. From the primary cohort enrolled between September 1, 2008 and June 30, 2011, 1,276 HD patients with an Hb measurement at enrollment were selected. All patients were included after obtaining written informed consent, and the Institutional Review Board of Kyungpook National University Hospital (2011-01-041) approved the study protocol. All clinical investigations were conducted according to the guidelines of the 2008 version of the Declaration of Helsinki.

### Patient demographic, clinical, and laboratory data and outcomes

Patient information, including basic demographic data, was recorded at enrollment; other clinical and biochemical variables were obtained every 6 or 12 months. Demographic and clinical data included age, sex, height, body weight, comorbidities, dialysis information, and medication, including the prescribed ESA dose and intravenous iron dose. Comorbidities included a history of diabetes, coronary artery disease, congestive heart failure, arrhythmia, stroke, or peripheral vascular disease.

Pre-dialysis venous blood samples were collected to establish the biochemical data, including Hb, serum albumin, iron status (serum iron, total iron binding capacity, and ferritin), and high-sensitivity C-reactive protein (hs-CRP). For calculating Kt/V, pre- and post-dialysis venous blood samples were obtained for serum urea nitrogen assessment. Hb was measured at baseline (enrollment), at 3 months subsequently, and every 6 months thereafter, while other laboratory variables were measured every 12 months. The ESAs used in this study were epoetin-α and darbepoetin. We used a conventional ratio of 200:1 to convert from epoetin-α to darbepoetin. For each patient, we used a weight-adjusted weekly ESA dose. The ESA responsiveness index (ERI) was calculated as previously defined: the dose of epoetin-α (IU/week) divided by Hb (g/dL) and dry weight (kg) [14].

The primary outcome measure was time to death from any cause. Date and cause of death were double-checked using data from Statistics Korea. Patient outcomes were assessed through December 31, 2011. The web-based system (http://webdb.crc-esrd.or.kr) was used for recording of data.

### Statistical analysis

All variables are represented as mean ± SD or number and percentage (%), depending on the nature of the variables. For comparison of values among groups, the chi-square test or one-way analysis of variance with Scheffé post-hoc test was used. The relationship between Hb and other explanatory variables including age, sex, diabetes, ESA dose, ferritin, serum albumin, Kt/V and hs-CRP was evaluated by simple regression analysis. For multiple regression analysis, variables were included as covariates, when they were significant in simple regression analysis. Cox proportional hazard analysis was conducted to estimate hazard ratios (HRs) and their 95% confidence interval (CI) for the association between the Hb level and ESA dose with mortality. Hb and ESA dose were treated as time-dependent variables measured every 6 months, and we made an imputation using the last observation carried forward method if the values were missed. Cox proportional hazard analysis was adjusted for all potential risk factors including age, sex, comorbidities, duration of dialysis, hs-CRP level, serum albumin level, ESA dose, serum ferritin level, and transferrin saturation. For further analysis, we stratified the patients by age or diabetes status, and the risk for mortality in association with Hb level was investigated by Cox proportional hazard analysis. All statistical analysis was performed using SAS system for Windows, version 9.2 (SAS Institute Inc., Cary, NC, USA), and R (R Foundation for Statistical Computing, Vienna, Austria; www.r-project.org). A two-sided P-value < 0.05 was defined as significant.

## Results

### Patient characteristics

The baseline characteristics of the 1,276 patients categorized using Hb levels (Hb groups) are shown in Table 1. The mean age of the patient population was 59.6 years. Men accounted for 58.4% of the sample, and 52.4% of patients had diabetes. At enrollment, the mean Hb of all the patients was 9.9 ± 1.7 g/dL. Stratification by Hb levels revealed that the ESA dose was significantly higher in the Hb group < 10 g/dL than that in the Hb group ≥ 10 g/dL. In addition, the ESA responsive index was the highest in the Hb group < 9 g/dL; further, it was significantly different as compared to that in the Hb group 9–10 g/dL and Hb group ≥ 10 g/dL. Serum ferritin and hs-CRP were also significantly higher in the Hb group < 9 g/dL as compared to the Hb group ≥ 9 g/dL. The percentage of patients receiving ESA treatment also varied significantly among the groups.

**Table 1:** Baseline characteristics of the cohort as classified by the levels of hemoglobin (Hb groups).

	All patients	Hb group					
	(n = 1276)	<9 (n = 72)	9–10 (n = 241)	10–11 (n = 421)	11–12 (n = 353)	12 (n = 189)	P
Age (years)	59.6 ± 13.8	58.9 ± 15.5	60.6 ± 12.8	60.3 ± 13.8	58.9 ± 14.2	58.0 ± 13.9	0.198
Male sex (%)	745 (58.4)	46 (63.9)	133 (55.2)	244 (58.0)	212 (60.1)	110 (58.2)	0.671
Body mass index (kg/m^2^)	22.4 ± 3.2	21.9 ± 2.8	22.1 ± 3.1	22.3 ± 3.4	22.8 ± 3.2	22.6 ± 2.9	0.071
History of diabetes (%)	536 (52.4)	28 (44.4)	100 (50.3)	181 (53.9)	146 (54.5)	81 (51.6)	0.598
History of CVD (%)							
Coronary artery disease	125 (12.3)	5 (8.1)	25 (12.6)	41 (12.3)	36 (13.6)	18 (11.5)	0.815
Congestive heart failure	115 (11.3)	8 (12.7)	25 (12.6)	40 (11.9)	25 (9.4)	17 (11.0)	0.814
Arrhythmia	19 (1.87)	1 (1.6)	2 (1.0)	8 (2.4)	5 (1.9)	3 (1.9)	0.862
Cerebrovascular disease	23 (2.3)	0 (0.0)	6 (3.0)	7 (2.1)	7 (2.6)	3 (1.9)	0.687
Peripheral vascular disease	63 (6.2)	2 (3.2)	12 (6.1)	23 (6.9)	17 (6.4)	9 (5.8)	0.858
ESA dose (U/week)*	8858.5 ± 5403.8	12595.5^b^ ± 8153.2	10760.2^b^ ± 6464.9	8140.9^a^ ± 4398.2	7647.8^a^ ± 4662.8	8555.6^a^ ± 4334.3	0.000
ERI (g/dL)−1/kg)*	14.9 ± 11.1	27.0^c^ ± 21.0	20.0^b^ ± 13.3	13.5^a^ ± 7.6	11.5^a^ ± 8.0	11.5^a^ ± 6.5	0.000
Serum albumin (g/dL)*	3.9 ± 0.4	3.7^a^ ± 0.4	3.8^b^ ± 0.4	4.0^c^ ± 0.4	4.0^c^ ± 0.4	4.0^c^ ± 0.4	0.000
Serum ferritin (ng/mL)*	275.5 ± 307.3	435.0^c^ ± 459.8	333.1^b^ ± 393.2	275.3^ab^ ± 295.6	226.7^a^ ± 211.2	237.8^a^ ± 264.0	0.000
TSAT (%)	30.6 ± 15.2	29.1 ± 15.1	30.7 ± 17.5	30.7 ± 15.1	30.9 ± 14.9	30.5 ± 12.3	0.949
hs-CRP (mg/L)*	0.7 ± 1.6	1.4^b^ ± 2.3	0.8^a^ ± 1.6	0.7^a^ ± 1.5	0.5^a^ ± 1.4	0.7^a^ ± 1.6	0.004
IV iron treatment (%)	99 (8.1)	11 (15.7)	22 (9.3)	27 (6.6)	28 (8.4)	11 (6.4)	0.078
ESA treatment** (%)	870 (71.8)	56 (82.4)	201 (85.9)	330 (80.9)	209 (62.4)	74 (44.6)	0.000
Kt/V (single pool)	1.4 ± 0.3	1.4 ± 0.2	1.4 ± 0.3	1.4 ± 0.2	1.4 ± 0.2	1.4 ± 0.3	0.199
Ca*(mg/dL)	8.7 ± 0.9	8.3^a^ ± 0.8	8.6^b^ ± 0.8	8.6^b^ ± 0.8	8.8^c^ ± 0.9	8.9^c^ ± 0.9	0.000
P*(mg/dL)	4.8 ± 1.5	4.7^ab^ ± 1.9	4.6^a^ ± 1.5	4.8^abc^ ± 1.5	5.0^bc^ ± 1.4	5.0^c^ ± 1.3	0.019

Continuous variables are noted as mean ± SD. Categorical variables are presented as n (%). CVD, cardiovascular diseases; ESA, erythropoiesis-stimulating agent; ERI, ESA responsiveness index; TSAT, transferrin saturation; hs-CRP, high-sensitivity C-reactive protein.

* Significant difference between a, b, and c (ANOVA, Scheffé test);

** significant difference among the five groups (Chi-square test).

### Predictors of Hb level

Table 2 shows the simple and multiple regression analysis between Hb levels and other biochemical variables. Hb levels showed a positive correlation with age, serum albumin and Kt/V but a negative correlation with serum ferritin and hs-CRP levels in simple regression analysis. After adjustment for age, ferritin, serum albumin, Kt/V and hs-CRP, age (β = 0.099; P < 0.001), serum albumin level (β = 0.409; P < 0.001) and Kt/V value (β = 0.209; P < 0.005) were independent predictors of higher Hb levels, while a higher serum hs-CRP level (β = -0.053; P = 0.056), with marginal significance, was an independent predictor of a lower Hb level on multiple regression analysis.

**Table 2:** Simple and multiple regression coefficients between hemoglobin and relevant variables at baseline.

Variable	Simple regression			Multiple regression		
	β	SE	*P*	β	SE	*P*
Age	0.063	0.003	0.024	0.099	0.003	0.000
Sex (Male)	-0.021	0.097	0.461			
DM	-0.033	0.097	0.248			
ESA dose	0.001	0.000	0.978			
Ferritin	-0.079	0.051	0.005	-0.045	0.048	0.105
Serum albumin	0.435	0.075	<0.001	0.409	0.084	0.000
Kt/V	0.185	0.207	<0.001	0.085	0.209	0.002
hs-CRP	-0.107	0.029	<0.001	-0.053	0.027	0.056

DM, diabetes mellitus; ESA, erythropoiesis-stimulating agent; hs-CRP, high-sensitivity C-reactive protein; Serum ferritin and high-sensitivity C-reactive protein (hs-CRP) levels were log-transformed. The multiple regression model included variables significant on simple analysis. Adjusted R-square is 0.199 (19%); β, coefficient; SE, standard error.

### Hb levels and mortality

The mean follow-up duration was 19.4 ± 8.5 months; in this period, 137 deaths (10.7%) occurred. Infectious diseases (28.5%) were ranked as the most common cause leading to death (Table 3).

**Table 3:** Causes of death.

Cause of death	Events (%)
Cardiovascular disease	19 (13.9)
Infectious disease	39 (28.5)
Cerebrovascular disease	5 (3.7)
Sudden death	17 (12.4)
Cancer	25 (18.3)
Other	14 (10.2)
Unknown	18 (13.1)
Total	137 (10.7)

Variables are noted as n (%).

**Fig 1:**
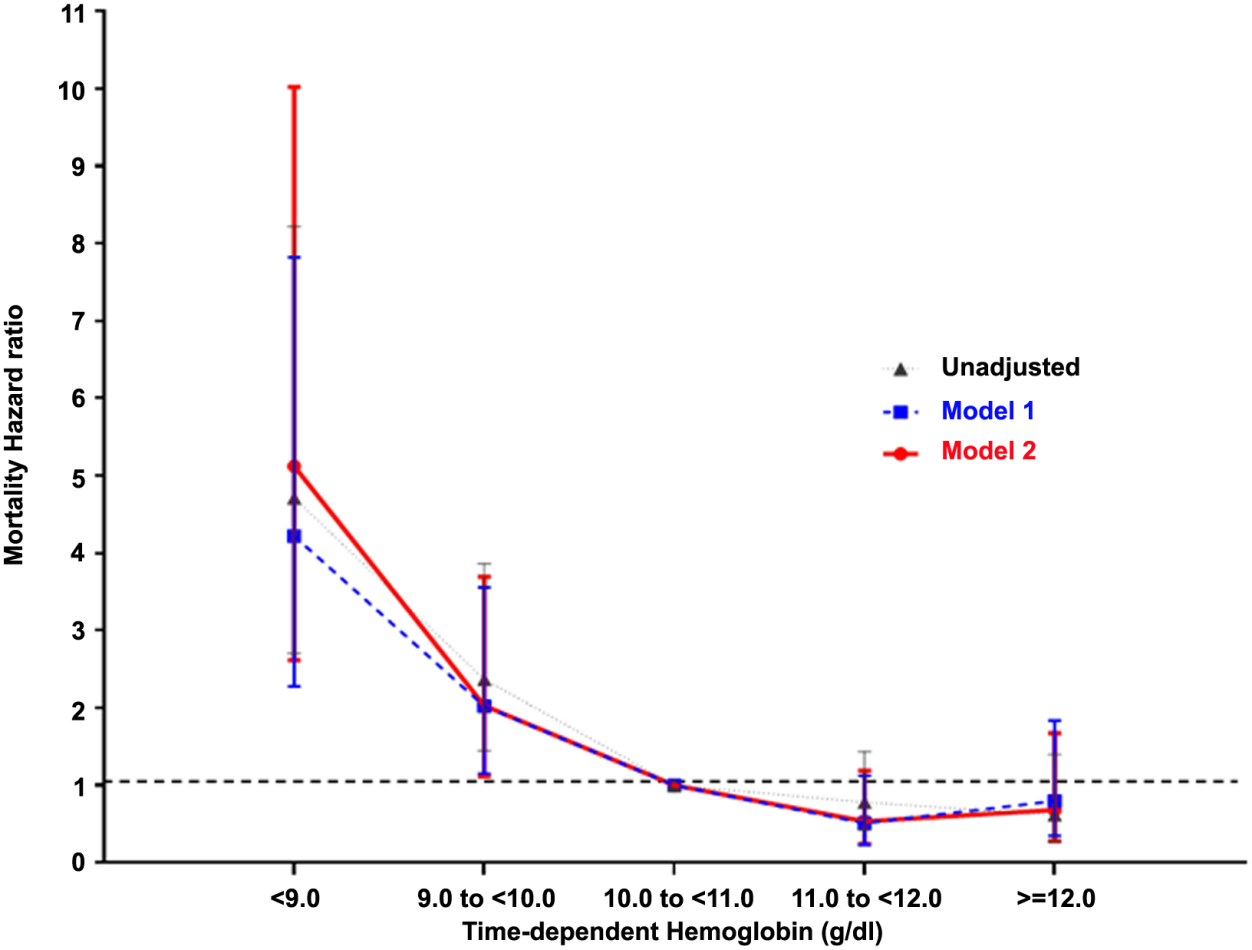
Hazard ratios of mortality based on time-dependent hemoglobin (Hb) levels in all the patients. Multivariate analysis sequentially adjusted models for covariates as follows. Model 1: age, sex, dialysis vintage, diabetes, cardiovascular disease, albumin, and high-sensitivity C-reactive protein; Model 2: Model 1 + erythropoiesis-stimulating agent (ESA) dose, ferritin, and transferrin saturation (TSAT). ESA dose was categorized as untreated (reference), 1 to <5000, 5000 to <10000, 10000 to <15000, and ≥15000 IU/week. Ferritin was categorized as <100, 100 to <500, and ≥500 ng/mL. TSAT was categorized as <20, 20 to <50, and ≥50%.

Fig 1 shows the HRs of mortality associated with time-dependent Hb levels using a reference Hb value of 10–11 g/dL. The unadjusted results showed significantly increased HRs (95% CI) for all categories of Hb group < 10.0 g/dL: 4.71 (CI, 2.70–8.22, P < 0.0001) and 2.37 (CI, 1.45–3.86, P < 0.0001) for Hb level < 9.0 and 9.0–10.0 g/dL, respectively. Multivariate Cox analysis showed increased HRs for Hb level < 10.0 g/dL, being 5.12 (CI, 2.62–10.02, P < 0.05) for Hb level < 9.0 g/dL and 2.03 (CI, 1.16–3.69, P < 0.05) for 9.0–10.0 g/dL. An Hb level ≥ 11 g/dL was not associated with a further decrease in HR for mortality (Table 4).

**Table 4:** Hazard ratios of mortality based on time-dependent Hb levels in all the patients.

Hb (g/dL)	Unadjusted		Model 1^a^		Model 2^b^		Number at risk, n (%)	Events, n (%)
	HR (95% CI)	P value	HR (95% CI)	P value	HR (95% CI)	P value		
<9.0	4.71 (2.70–8.22)	<0.0001	4.22 (2.28–7.82)	<0.0001	5.12 (2.62–10.02)	<0.0001	90 (7.05)	20 (22.2)
9.0 to <10.0	2.37 (1.45–3.86)	0.0006	2.02 (1.15–3.55)	0.0145	2.03 (1.16–3.69)	0.0206	217 (17.01)	35 (16.1)
10.0 to <11.0	Reference		Reference		Reference		496 (38.87)	36 (7.3)
11.0 to <12.0	0.78 (0.42–1.43)	0.4216	0.51 (0.23–1.12)	0.0943	0.54 (0.24–1.19)	0.1250	292 (22.88)	16 (5.5)
≥12.0	0.62 (0.27–1.39)	0.2455	0.80 (0.35–1.83)	0.5927	0.68 (0.28–1.67)	0.4015	181 (14.18)	9 (5.0)

Hb, hemoglobin; HR, hazard ratio; CI, confidence interval

Multivariate analysis sequentially adjusted models for covariates as follows:

^a^Model 1: age, sex, dialysis vintage, diabetes, cardiovascular disease, albumin, and high-sensitivity C-reactive protein

^b^Model 2: Model 1 + erythropoiesis-stimulating agent (ESA) dose, ferritin, and adjusted transferrin saturation (TSAT). Hazard ratios given for a 10-unit increase in age and two-unit increase in dialysis vintage.

ESA dose was categorized as untreated (reference), 1 to <5000, 5000 to <10000, 10000 to <15000, and ≥15000 IU/week.

Ferritin was categorized as <100, 100 to <500, and ≥500 ng/mL. TSAT was categorized as <20, 20 to <50, and ≥50.

**Fig 2:**
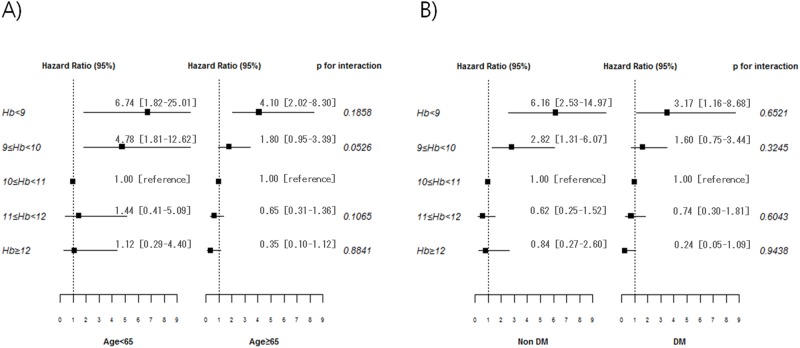
Hazard ratios of mortality based on time-dependent Hb level according to the age group (A) and diabetes status (B). Multivariate analysis used covariates as follows: age, sex, dialysis vintage, diabetes, cardiovascular disease, albumin, and high-sensitivity C-reactive protein, erythropoiesis-stimulating agent (ESA) dose, ferritin, and transferrin saturation (TSAT). HR, hazard ratio; CI, confidence interval

To evaluate the effect of age on the association of Hb level and mortality, we divided the patients into older and younger groups based on the age of 65 years, and performed Cox proportional hazard analysis with time-dependent Hb levels. In the younger group, HRs were 6.74 (CI, 1.82–25.01, P < 0.05) for Hb level < 9.0 g/dL and 4.78 (CI, 1.81–12.62, P < 0.05) for Hb 9.0–10.0 g/dL. However, risk for mortality increased only at Hb level < 9.0 g/dL in the older group (HR 4.10, CI, 2.02–8.30, P < 0.005). The interaction of Hb and age was not statistically significant at Hb level < 9.0 g/dL, whereas there was marginal significance of interaction at Hb level 9.0–10.0 g/dL (P for interaction = 0.0526) (Fig 2A). In other words, HRs for mortality tended to be higher in the younger group compared to the older group only in Hb category 9–10 g/dL. Moreover, we evaluated the effect of diabetes status on the association of Hb and mortality. In Cox proportional hazard analysis, non-diabetic patients had a higher risk for mortality in both Hb categories (HR 6.16, CI, 2.53–14.97, P < 0.001 at Hb < 9.0 g/dL; HR 2.82, CI, 1.31–6.07, P < 0.05 at Hb 9.0–10.0 g/dL, respectively), whereas diabetic patients had a higher risk for mortality only in Hb category < 9.0 g/dL (HR 3.17, CI, 1.16–8.68, P < 0.05). However, the interaction of diabetes status and mortality was not statistically significant in either Hb category of < 9.0 or 9.0–10.0 g/dL (P = 0.6521 and P = 0.3245, respectively) (Fig 2B). This suggests HRs for mortality were not different between diabetics and non-diabetics at Hb level < 10 g/dL.

### ESA dose and mortality

**Fig 3:**
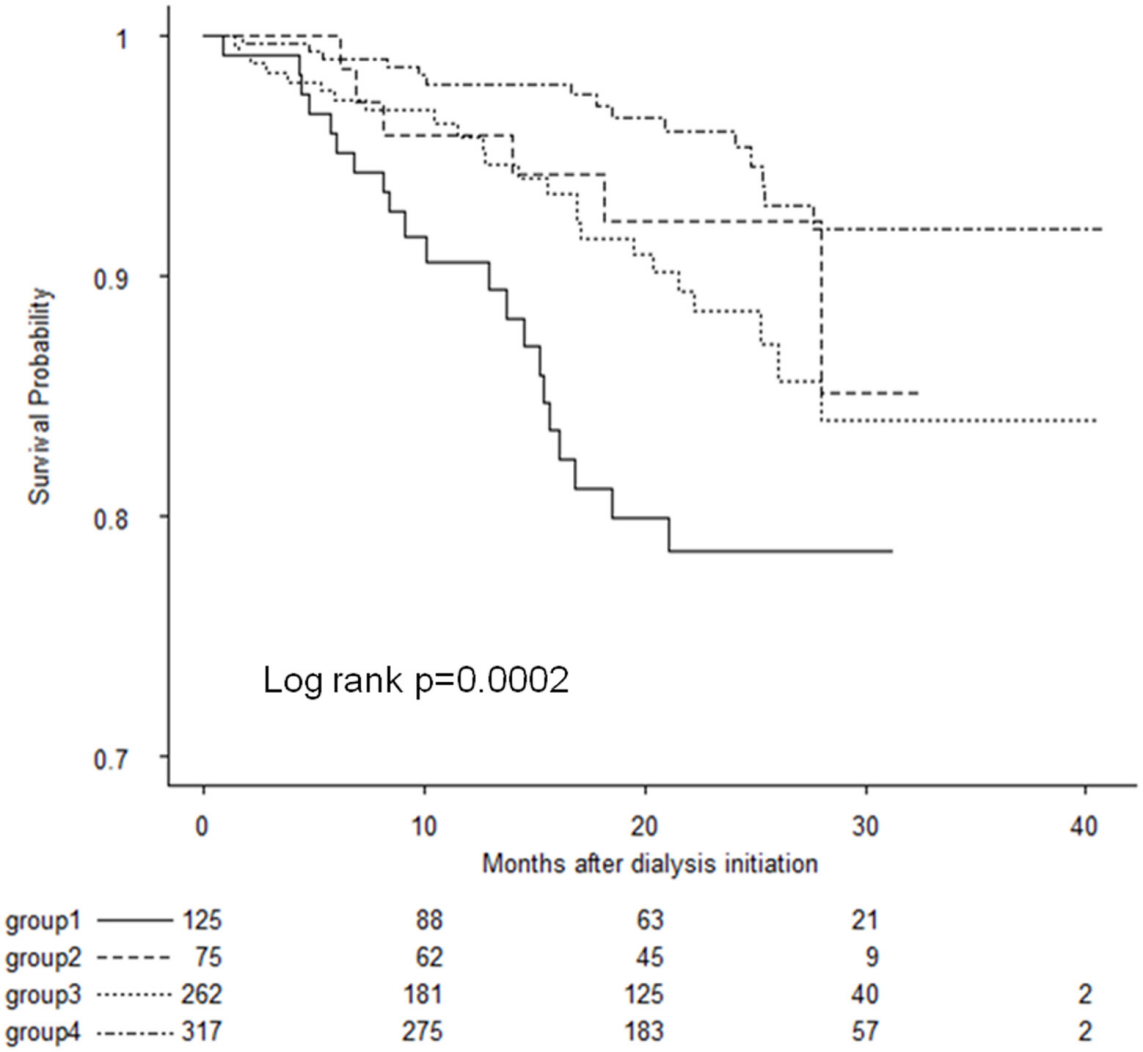
Survival probability according to time-dependent Hb and time-dependent erythropoiesis-stimulating agent dose categories in all the patients. Group 1, Hb < 10 g/dL and ESA ≥ 126 U/week/kg; group 2, Hb < 10 g/dL and ESA < 126 U/week/kg; group 3, Hb ≥ 10 g/dL and ESA ≥ 126 U/week/kg; and group 4, Hb ≥ 10 g/dL and ESA < 126 U/week/kg.

Next, we evaluated whether the ESA dose affected mortality risk based on the Hb levels in all the patients. With reference to an Hb level of 10 g/dL and a median weekly ESA dose of 126 U/week/kg, we divided all the patients into four groups: group 1, Hb < 10 g/dL and ESA ≥ 126 U/week/kg; group 2, Hb < 10 g/dL and ESA < 126 U/week/kg; group 3, Hb ≥ 10 g/dL and ESA ≥ 126 U/week/kg; and group 4, Hb ≥ 10 g/dL and ESA < 126 U/week/kg. In each Hb category, high-dose ESA was associated with an increased risk of mortality, and this association was statistically significant in the groups with Hb level < 10 g/dL. Survival analysis using the Kaplan-Meier curve with log-rank test revealed that the group 1 with Hb level < 10 g/dL with higher ESA (≥126 U/week/kg) had the lowest survival (Fig 3). Using both time-dependent Hb levels and ESA dose, the unadjusted and adjusted HRs for mortality were 3.55 (CI, 1.67–7.55, p = 0.001) and 2.25 (CI, 1.03–4.92 p = 0.043), respectively, in the group 1 as compared to the reference group with an Hb level of 10–11 g/dL and ESA dose < 126 U/week/kg (Table 5).

**Table 5:** Hazard ratios of mortality based on the hemoglobin level and ESA dose in all the patients.

Hb (g/dL)	ESA (U/week/kg)	Unadjusted model		Model 1^a^		Model 2^b^		Number at risk, n (%)	Events, n (%)
		HR (95% CI)	P value	HR (95% CI)	P value	HR (95% CI)	P value		
<10.0	<126	1.49 (0.54–4.09)	0.4427	1.13 (0.40–3.15)	0.8179	0.99 (0.35–2.78)	0.9876	79 (9.48)	6 (7.59)
	≥126	3.55 (1.67–7.55)	0.0010	2.53 (1.16–5.53)	0.02	2.25 (1.03–4.92)	0.0430	138 (16.57)	24 (17.39)
10.0 to <11.0	<126	1.00 (Reference)		1.00 (Reference)		1.00 (Reference)		193 (23.17)	10 (5.18)
	≥126	2.38 (1.11–5.09)	0.0252	2.12 (0.99–4.58)	0.05	1.89 (0.87–4.09)	0.1072	188 (22.57)	22 (11.70)
≥11.0	<126	0.69 (0.23–2.00)	0.4895	0.55 (0.19–1.63)	0.28	0.42 (0.13–1.36)	0.1495	144 (14.29)	5 (3.47)
	≥126	1.05 (0.33–3.36)	0.9294	1.06 (0.33–3.42)	0.93	1.01 (0.31–3.26)	0.9891	91 (10.92)	4 (4.40)

ESA, erythropoietin-stimulating agent; Hb, hemoglobin; HR, hazard ratio; CI, confidence interval

Multivariate analysis sequentially adjusted models for covariates as follows:

^a^Model 1: age, sex, dialysis vintage, diabetes, cardiovascular disease, albumin, high-sensitivity C-reactive protein

^b^Model 2: Model 1 + ferritin and adjusted transferrin saturation (TSAT). Hazard ratios given for a 10-unit increase in age and two-unit increase in dialysis vintage. An Hb level of 10–11 g/dL and ESA dose < 126 U/week/kg were used as the reference.

## Discussion

In this analysis of 1,276 HD patients from a Korean nationwide prospective cohort, we found that a time-dependent low Hb level (<10 g/dL) was consistently associated with high mortality after adjustment for age, sex, comorbidities, intravenously injected iron, ESA dose, iron status, and nutritional and inflammatory status. Furthermore, we found no apparent benefit in survival rate in patients with Hb levels > 11 g/dL compared to those with an Hb level of 10–11 g/dL. Regarding ESA dose, the mortality risk was the highest in patients with an Hb level < 10 g/dL and a higher dose of ESA (≥126 U/week/kg).

Although the association between low Hb and mortality risk has been well known in HD patients, the appropriate Hb target has remained under debate. Several observational studies have demonstrated an association between low Hb level and mortality in HD patients [3, 5, 13, 15, 16]. Regidor *et al*. [13] reported that a time-dependent Hb level < 11.5 g/dL was associated with an increased risk of death in a 2-year cohort of 58,058 HD patients after adjustment for several risk factors including inflammation, nutritional parameters, and status of iron and medications (intravenous iron and ESA doses). In a recent report of 2,310 incident HD patients in Spain, an association was observed between Hb level ≤ 10.0 g/dL and increased risk of mortality after adjustment by multivariate analysis [16].

In the present study, a low Hb level was associated with an increased risk of mortality in Korean HD patients. Specifically, patients with an Hb level < 10 g/dL showed a significantly increased mortality risk. Patients with a time-dependent Hb level < 9.0 g/dL and an Hb level of 9.0–10.0 g/dL had a 5.1- and 2.0-fold higher risk of death, respectively, compared to those with an Hb level of 10–11 g/dL. However, patients with a time-dependent Hb level ≥ 11.0 g/dL had no additional survival advantage compared to those with an Hb level of 10–11 g/dL after adjustment for covariates. This result is consistent with other studies from Asian countries. A Japanese study has shown that in non-elderly patients, an Hb level < 10 g/dL increased the risk of patient mortality as compared to an Hb level of 10–11 g/dL, whereas an elderly population were at significant risk with an Hb level < 9 g/dL [14]. In addition, a recent Taiwanese study suggested a target Hb level 10–11 g/dL while avoiding disproportionately high ESA doses [17]. In contrast, in a Spanish study of incident HD patients, the mortality risk was lower in patients with Hb level > 13 g/dL compared to patients with a reference Hb level of 11–12 g/dL, regardless of ESA dose. They showed that the mortality risk did not increase even at high ESA doses (>16000 U/week) [18].

It is difficult to draw a conclusion simply based on these observational studies. Several RCTs have suggested that more intensive treatment aimed at normalizing Hb using higher doses of ESA appears to be more harmful than beneficial [6–9]. The Normal Hematocrit Cardiac Trial of 1,265 HD patients demonstrated that a higher Hb target (13–15 g/dL) was associated with an increased risk of mortality compared with a lower Hb target (9–10 g/dL) [6, 19]. However, differences in Hb levels or a higher ESA dose could not explain the difference in mortality observed between the two groups in this study [6]. The median achieved Hb concentration was 12.6 g/dL in the higher target Hb group and 10.3 g/dL in the lower target Hb group, while the mortality rates decreased with increasing Hb values in both groups [6]. Moreover, the Correction of Hemoglobin and Outcomes in Renal Insufficiency (CHOIR) trial showed that a higher Hb target of 13.5 g/dL was associated with an increased risk of mortality than a lower Hb target of 11.3 g/dL. Mean doses of epoetin-α were 1.8-fold higher in the higher Hb target group [8]. Similarly, the Trial to Reduce cardiovascular Events with Aranesp Therapy reported that the use of darbepoetin-α (achieved Hb of 13.0 g/dL) did not reduce the risk of overall mortality or cardiovascular events but rather was associated with an increased risk of stroke as compared to the placebo arm treated with rescue darbepoetin-α at an Hb < 9.0 g/dL [9].

The reasons underlying the poor outcomes associated with higher Hb targets in these RCTs are yet to be elucidated. Secondary analysis of the CHOIR trials demonstrated that inability to achieve target Hb and the use of high-dose ESA were associated with poorer outcomes [20]. Zhang *et al*. [21] reported that a patient receiving a higher dose of ESA had lower Hb levels and greater mortality in a retrospective cohort study of HD patients. Similar results were reported by Japan’s dialysis registry [22]. In that study, investigators defined ESA hyporesponsiveness as Hb level < 10 g/dL at an ESA dosage ≥ 6,000 U/week; they reported that mortality risk can be affected by ESA dose and Hb level in an interactive way [22].

In the present study, the weight-adjusted median weekly ESA dose converted to epoetin-α was 126 U/kg/week. The group with lower Hb levels but higher ESA doses was at an increased risk of death than those with an Hb level of 10–11 g/dL and a lower ESA dose. In a recent meta-analysis of data from 31 trials, a higher ESA dose was associated with all-cause mortality regardless of the Hb level [23]. A higher ESA dose is associated with an increased rate of adverse events, including hypertension, stroke, and thrombotic events. A higher ESA dose reflects patients’ comorbidities, malnutrition-inflammation syndrome, or hypercatabolism. In the present study, we also investigated the clinical factors affecting Hb levels; accordingly, better nutritional status and dialysis adequacy were associated with higher Hb levels, while a higher inflammatory status was associated with lower Hb levels. These findings suggest that higher Hb levels are difficult to achieve in patients with poor nutritional status, low dialysis adequacy, and higher inflammatory status even with higher ESA doses.

We also investigated whether the survival benefit differs by patient characteristics, including patient age or the presence of diabetes. In subgroup analysis stratified by age or diabetes status, HR for mortality was significantly increased in Hb category < 9 g/dL in both older and younger groups, whereas in Hb category 9–10 g/dL, the increased risk for mortality was statistically significant only in the younger group. However, the interaction of age and Hb level on mortality risk tended to be significant only at Hb level 9–10 g/dL. This result suggests that the older group might tolerate lower Hb better than the younger group, especially at Hb level 9–10 g/dL. Similar to our findings, the Japan Dialysis Outcomes and Practice Patterns Study (J-DOPPS) cohort demonstrated that the population aged over 75 years had poorer prognosis only at Hb < 9 g/dL [14]. The marginal significance of interaction in our study might be related to the smaller number of patients compared to the Japanese study.

Regarding the effect of diabetes on the association of Hb and mortality, our results demonstrated that increased risk for mortality was significant in Hb category < 9 g/dL in both diabetics and non-diabetics, whereas in Hb category 9–10 g/dL the risk for mortality increased only in non-diabetics. The HRs were higher in non-diabetics compared to diabetics, although we could not identify a significant interaction between diabetes status and Hb level. This might be because other overwhelming poor prognostic factors masked or attenuated the prognostic effect of lower Hb on mortality. A secondary analysis of the CHOIR trial has also shown that diabetes status did not interact with Hb target in Cox proportional hazard analysis [24]. However, a previous study revealed that the association of Hb level and mortality was lost in diabetic HD patients [25]. In addition, a study from J-DOPPS has also demonstrated that interaction between age and Hb was lost in diabetic patients [14].

This study has certain limitations. First, it was an observational study; therefore, we had limited information on the cause and effect relationship between the Hb level or ESA dose and mortality. Second, it did not include the patients’ histories of blood transfusion, which would be an important factor affecting Hb level and mortality. Third, due to limitations of our subgroup analysis that investigated whether the survival benefit differs by patient characteristics, including the presence of diabetes and patient age, and the lower statistical power in the interaction of age or diabetes status and Hb, one should be very cautious in determining Hb target according to patient characteristics. Fourth, we could not exclude the possible effect of residual confounding factors affecting the association of Hb and mortality, even though we tried to adjust for this by using multiple covariates. Finally, several questions remain about whether this Hb target of 10–11 g/dL affects the transfusion rate, quality of life, or cardiovascular safety improvements.

Nevertheless, our study also has several strengths. First, it analyzed prospective data, including a full range of demographic and laboratory parameters of >1,000 patients in 31 centers across Korea rather than a single for-profit dialysis registry. Second, we analyzed time-dependent variables to which longitudinal variability may be attributed.

In conclusion, the Hb level was correlated with dialysis adequacy, malnutrition, and inflammation. A low Hb level was a significant predictor of mortality in HD patients independent of comorbidity, malnutrition, inflammation, and iron status. Using CRC-ESRD data, we validated the association between Hb levels/ESA dose and mortality in Korean HD patients. The clinical practice target of an Hb level of 10–11 g/dL before the new KDIGO anemia guideline era appears reasonable, considering the survival benefit in HD patients in this Korean prospective observational cohort.
